# About the activity and selectivity of less well-known metathesis catalysts during ADMET polymerizations

**DOI:** 10.3762/bjoc.6.131

**Published:** 2010-12-03

**Authors:** Hatice Mutlu, Lucas Montero de Espinosa, Oĝuz Türünç, Michael A R Meier

**Affiliations:** 1 University of Potsdam, Institute of Chemistry, Karl-Liebknecht-Str. 24-25, 14476 Golm, Germany; 2 University of Applied Sciences Emden/Leer, Constantiaplatz 4, 26723 Emden, Germany; 3 Max-Planck-Institute of Colloids and Interfaces, Department of Colloid Chemistry, Potsdam, Germany

**Keywords:** ADMET, metathesis, olefin isomerization, renewable raw materials, ruthenium–indenylidene catalysts

## Abstract

We report on the catalytic activity of commercially available Ru-indenylidene and “boomerang” complexes **C1**, **C2** and **C3** in acyclic diene metathesis (ADMET) polymerization of a fully renewable α,ω-diene. A high activity of these catalysts was observed for the synthesis of the desired renewable polyesters with molecular weights of up to 17000 Da, which is considerably higher than molecular weights obtained using the same monomer with previously studied catalysts. Moreover, olefin isomerization side reactions that occur during the ADMET polymerizations were studied in detail. The isomerization reactions were investigated by degradation of the prepared polyesters via transesterification with methanol, yielding diesters. These diesters, representing the repeat units of the polyesters, were then quantified by GC-MS.

## Introduction

Among the large number of organic and organometallic reactions allowing the formation of carbon–carbon bonds, olefin metathesis has found its place in organic synthesis as well as polymer science as a very versatile tool that allows transformations that were previously not (or hardly) possible [1–6]. This academic and industrial success is also closely associated with the development and commercialization of efficient catalysts.

**Figure 1:**
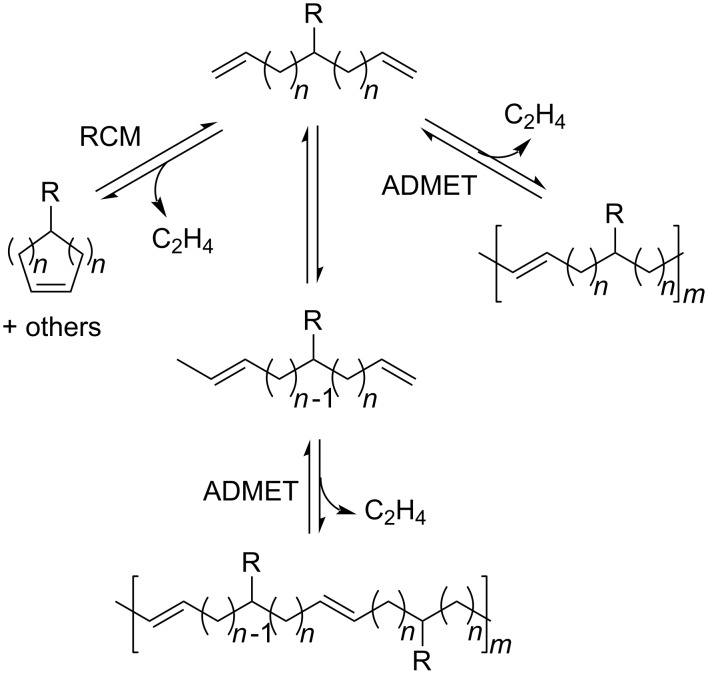
Olefin isomerization during ADMET polymerization.

In the past few years, researchers realized that olefin isomerization is an important side reaction of Ru-catalyzed metathesis reactions. First reports on olefin isomerization claimed that this undesired side reaction was observed on substrates containing allylic oxygen or nitrogen functional groups in combination with first generation catalysts [7–11]. Later it was demonstrated that the degradation product of Grubbs 1^st^ generation catalyst was capable of catalyzing olefin isomerization [12]. Double bond isomerization was also observed with 2^nd^ generation catalysts on a broad variety of substrates competitively and sometimes prior to olefin metathesis [13–17]. In a number of other publications this problem was addressed and further discussions on the possible mechanism of the two proposed pathways, the π-allyl metal hydride and the metal hydride addition-elimination mechanisms, were reported [8,11,13–18]. In most cases isomerization was attributed to the presence of a Ru-hydride species [13–14]. The cause of formation of such Ru-hydride species was long a subject of discussion. Grubbs reported that certain ruthenium carbene complexes can thermally decompose to Ru-hydride species [19]. Moreover, mechanistic investigation of the thermal decomposition of the Grubbs second generation catalyst carried out by Grubbs and co-workers clearly showed that prolonged heating of the catalyst results in the formation of a binuclear ruthenium hydride complex [20]. The observation that this binuclear product was capable of efficiently isomerizing terminal olefins is a clear indication that metal hydride species are indeed the source of the isomerization. It was reported that a proper selection of solvents and additives can eliminate isomerization with Ru-based metathesis catalysts in RCM [16]. The addition of POCy_3_ or oxygen inhibits isomerization, whereas the use of more coordinating solvents favors it. Additional research in this area reported that other types of additives, such as acetic acid [21], chlorocatecholborane [22], boron-based Lewis acid (such as: Cy_2_BCl) [23], or PhOP(O)(OH)_2_ [24] can reduce the isomerization activity of the catalyst. Furthermore, Johnson and coworkers reported that during a RCM to make a 9-membered ring, chlorinated solvents, such as 1,2-dichloroethane, inhibited olefin isomerization [25]. Grubbs and collaborators showed that catalytic amounts (10 mol %) of 1,4-benzoquinone (**BQ**) can prevent the isomerization of a number of allylic ethers and long chain aliphatic alkenes during RCM and cross metathesis [21]. In the context of ADMET, isomerization of a terminal to an internal olefin, followed by a productive metathesis step with a terminal olefin, would liberate an α-olefin, such as propene or 1-butene, as opposed to the ethylene liberated from a conventional ADMET reaction of two terminal olefins (Figure 1) [26]. Release of these higher condensate molecules would decrease the mass yield of the polymer, and if olefin isomerization occurs in a similar timescale as metathesis, this would result in polymers with ill-defined repeat units, which would also affect the physical properties of the polymer. Noteworthy, under ADMET conditions, the first-generation Ru-catalyst was found not to isomerize olefins [27].

In model studies carried out with simple olefins, Wagener and co-workers demonstrated that, while Grubbs 1^st^ generation and Schrock's molybdenum alkylidene catalysts did not produce appreciable double bond isomerization, Grubbs 2^nd^ generation catalyst presented significant isomerization activity, which was greatly reduced at temperatures below 30 ºC [17,28]. These studies were further complemented and confirmed by MALDI analysis of an amino acid polymer synthesized with Grubbs 2^nd^ generation catalyst [29].

Recently, a detailed study of temperature, catalyst, and polymerization condition dependent isomerization side reactions that occur during ADMET polymerizations was reported by Meier and Fokou [27]. The study clearly showed that high temperatures, such as 100 °C, increased the amount of isomerization for Grubbs 2^nd^ generation catalyst. In order to better understand the behavior of several second generation metathesis catalysts under ADMET conditions, their isomerization tendencies were subsequently studied [30]. The investigated catalysts showed high degrees of isomerization at 80 °C. The addition of **BQ** provided the best results in terms of reducing the isomerization reactions when added prior to the catalyst, indicating that catalyst decomposition begins as soon as the catalyst is added to the reaction mixture at high reaction temperatures. The effects of nitrogen purging and higher temperatures in the presence of **BQ** were also investigated and revealed that with nitrogen purging the degree of isomerization remained similar or even decreased.

**Figure 2:**
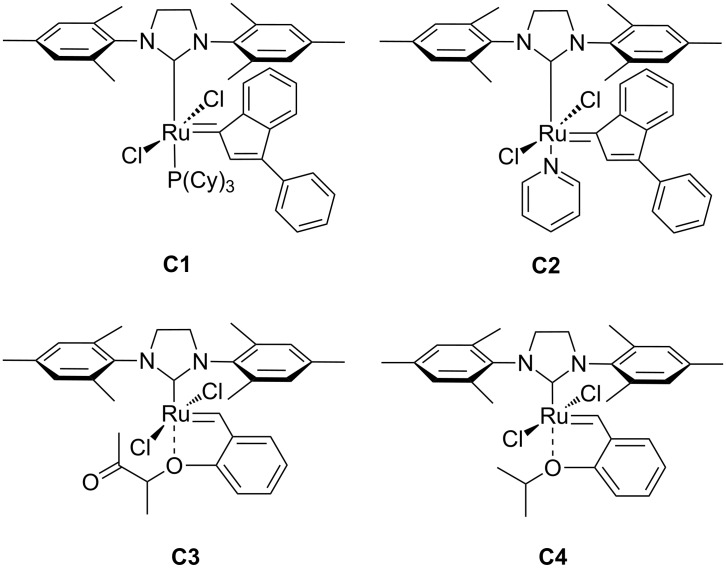
Ru–indenylidene metathesis catalysts **C1** and **C2**, “boomerang” complexes **C3,** and Hoveyda–Grubbs 2^nd^ generation catalyst **C4** were studied for their efficiency and isomerization tendency in ADMET polymerizations.

Among the numerous metathesis initiators available, we focused this study on the application of the less investigated indenylidene Ru-based catalysts: (1,3-bis(2,4,6-trimethylphenyl)-2-imidazolidinylidene) dichloro-(3-phenyl-1*H*-inden-1-ylidene)(tricyclohexylphosphine) ruthenium(II) (**C1**), (1,3-bis(2,4,6-trimethylphenyl)-2-imidazolidinylidene)dichloro-(3-phenyl-1*H*-inden-1-ylidene)(pyridyl) ruthenium(II) (**C2**) and the newly developed “boomerang” complex (1,3-bis(2,4,6-trimethylphenyl)-2-imidazolidinylidene)dichloro(2-(1-methylacetoxy)phenyl)methylene ruthenium(II) (**C3**) [31] (Figure 2).

These indenylidene Ru-complexes provide an attractive alternative to the Ru–benzylidene compounds. It was shown that all indenylidene Ru-catalysts were more robust under the demanding reaction conditions (temperature and functional group tolerance) compared to their Ru–benzylidene counterparts [32–40]. In addition, good catalytic activities in RCM of linear dienes [32,34–35] and ROMP of cycloolefins [36–40] were reported. RCM studies with diethyl diallylmalonate and diallyl tosylamine as substrates showed an appreciable catalytic activity and selectivity for the 2^nd^ generation 16-electron Ru–indenylidene complex (**C1**) [41]. High temperatures allow for better ligand dissociation, and hence for a higher initiation rate of **C1** in RCM [33,35]. Moreover, good activities were obtained in the self-metathesis reaction of undecylenic aldehyde, a renewable building block derived from castor oil cracking [42]. Research performed by Monsaert et al. illustrated that **C2** enables high conversions in ROMP of 1,5-cyclooctadiene, and conversions of up to 80% in the RCM of diethyl diallylmalonate in short reaction times (5–10 min), thus being superior to the benzylidene analogue [35].

Recently, a useful and practical guide to application of olefin metathesis catalysts was published by Grela and co-workers [43]. They examined the effectiveness of Ru–indenylidene complexes in standard olefin metathesis reactions and compared their activities to those of Grubbs and Hoveyda–Grubbs type catalysts. In contrast to Grubbs and Hoveyda–Grubbs catalysts, **C1** was found to be practically inactive toward the RCM of diethyl diallymalonate at room temperature with catalyst loadings as low as 0.05 mol %. However, conversions dramatically increased when the reaction temperature was increased to 70 °C. In addition, application of **C1** to challenging substrates, such as diethyl di(methallyl)malonate in ﬂuorinated aromatic hydrocarbon solvents, resulted in a remarkable enhancement of catalytic activity. Moreover, this approach was successfully extended to the RCM of natural products and the cross-metathesis formation of trisubstituted alkenes [44].

Thus, we decided to study the catalytic activity of **C1**, **C2** and **C3** in ADMET polymerizations. Furthermore, to gain insight into isomerization activities of the catalysts, detailed isomerization studies were also performed using a procedure already described in the literature [30]. The catalyst loading (0.5 mol %) was kept constant throughout the entire screening process and temperatures varied from 60 °C to 120 °C during the investigation.

## Results and Discussion

**Figure 3:**
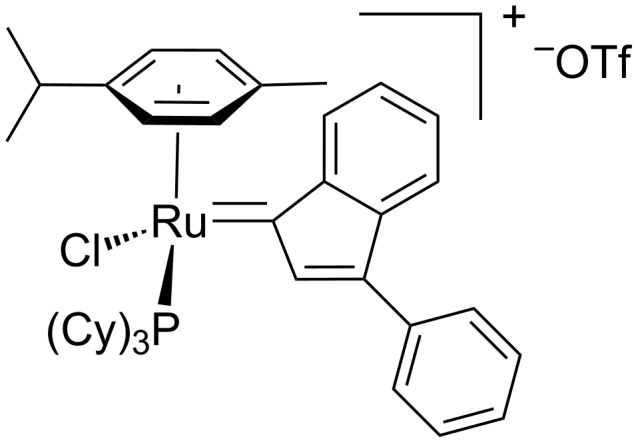
Representative scheme for the in situ generated Ru–indenylidene [38].

To date, only one example of ADMET polymerization with an in situ generated Ru–indenylidene catalyst has been reported [38]. The related arene Ru–indenylidene complex (Figure 3) was generated in situ from [RuCl(*p*-cymene)(=C=C=CPh_2_)(PCy_3_)][CF_3_SO_3_], as the catalyst precursor and HOSO_2_CF_3_, and applied in the ADMET of 1,9-decadiene to yield a polymer with 94% conversion in 12 h at 0 °C.

**Figure 4:**
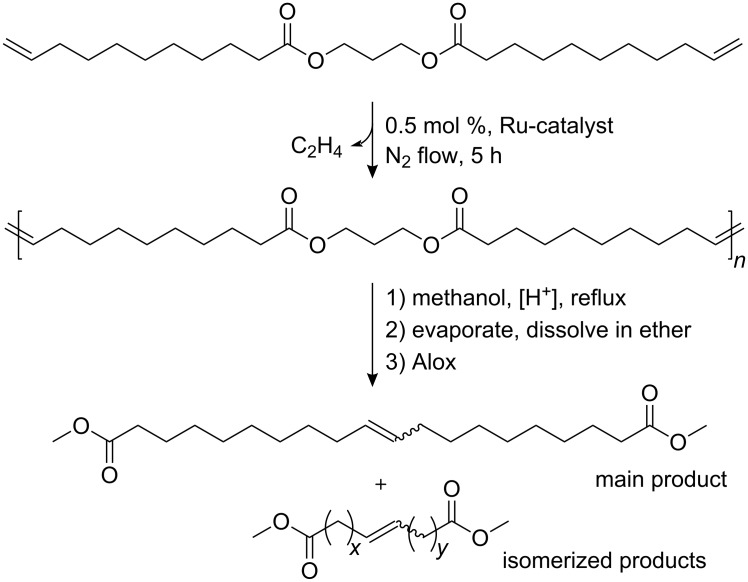
Synthesis of the studied α,ω-diene, its ADMET polymerization, and the strategy to evaluate isomerization side reactions.

In this contribution, we report for the first time on the performance of two well-defined, stable Ru–indenylidene catalysts **C1** and **C2**, and the “boomerang complex” **C3** (Figure 2) during ADMET polymerizations. The ADMET monomer was synthesized by a procedure adapted from the literature using 1,3-propanediol, which can be prepared from glycerol, and 10-undecenoic acid [45], a commercial derivative of castor oil (Figure 4). A set of ADMET polymerizations was used to evaluate the performance of complexes **C1**, **C2** and **C3** at four different temperatures (60, 80, 100 and 120 °C), under bulk conditions, after 5 h reaction time, and constant catalyst loading (200:1 = monomer **1**: catalyst). This provided a broad data set to screen the catalytic systems tested (Table 1 and Table 2). The activity of these catalysts was compared to the Hoveyda–Grubbs 2^nd^ generation catalyst (**C4**), which was previously examined in ADMET polymerizations of the same monomer [30]. In all cases, continuous nitrogen purging was applied throughout the polymerizations and polymerizations were run in duplicate to obtain a reliable set of data.

**Table 1:** Overview of polymerization and the isomerization results of the corresponding polymers obtained at 60 and 80 °C after 5 h reaction time.

Entry	Polymer	Cat % [0.5 mol %]	Temp °C	Conditions^a^	Iso %^b^	*M*_n_ (Da)^c^	PDI

1	**P1**	**C1**	60		36.3	10500	2.00
2	**P2**	**C1**	60	**BQ** [1%]	0.70	8300	2.05
3	**P3**	**C2**	60		9.91	1700	1.16
4	**P4**	**C2**	60	**BQ** [1%]	NI^d^	2200	1.36
5	**P5**	**C3**	60		69.6	8000	1.60
6	**P6**	**C3**	60	**BQ** [1%]	63.9	4200	1.76
7	**P7**	**C1**	80		63.9	14000	1.92
8	**P8**	**C1**	80	**BQ** [1%]	74.2	14000	2.09
9	**P9**	**C2**	80		41.9	14200	1.90
10	**P10**	**C2**	80	**BQ** [1%]	28.6	9200	1.90
11	**P11**	**C3**	80		91.4	11850	1.80
12	**P12**	**C3**	80	**BQ** [1%]	59.2	11300	1.93

^a^Additional conditions applied during polymerization: **BQ**: amount of benzoquinone in % with respect to monomer; ^b^% amount of isomerized diesters observed with GC-MS after transesterification of the respective polymer; ^c^GPC was performed in THF, containing BHT, with PMMA calibration; ^d^NI: no isomerization.

**Table 2:** Overview of polymerization and the isomerization results of the corresponding polymers obtained at 100 and 120 °C after 5 h reaction time.

Entry	Polymer	Cat % [0.5 mol %]	Temp °C	Conditions^a^	Iso %^b^	*M*_n_ (Da)^c^	PDI

13	**P13**	**C1**	100		79.3	10000	1.79
14	**P14**	**C1**	100	**BQ** [1%]	81.6	11300	1.74
15	**P15**	**C2**	100		53.6	9000	1.85
16	**P16**	**C2**	100	**BQ** [1%]	0.80	4500	1.60
17	**P17**	**C3**	100		55.2	6700	1.72
18	**P18**	**C3**	100	**BQ** [1%]	37.2	10150	1.92
19	**P19**	**C1**	120		89.4	16700	1.80
20	**P20**	**C1**	120	**BQ** [1%]	73.0	11000	1.83
21	**P21**	**C2**	120		83.7	13000	1.66
22	**P22**	**C2**	120	**BQ** [1%]	16.0	8500	1.78
23	**P23**	**C3**	120		87.4	12200	1.73
24	**P24**	**C3**	120	**BQ** [1%]	73.8	14850	1.73
25	**P25**	**C4**	120		80.5	10400	1.93
26	**P26**	**C4**	120	**BQ** [1%]	66.5	12000	1.67

^a^Additional conditions applied during polymerization: **BQ**: amount of benzoquinone in % respective to monomer; ^b^% amount of isomerized diesters observed with GC-MS after transesterification of the respective polymer ^c^GPC was performed in THF, containing BHT, with PMMA calibration..

Moreover, the resulting ADMET polymers were transesterified with methanol to yield α,ω-diesters, which were subsequently analyzed by GC-MS (Figure 4). For the polymerizations in which isomerization does not occur, the GC-MS would only show a single peak corresponding to the unsaturated C-20 repeating unit of the studied polymers (compare Figure 4). However, most ruthenium-based metathesis catalysts are known to promote olefin isomerization. As a result, the corresponding transesterified polymer yields a mixture of diesters with different chain lengths, since double bond isomerisation and olefin metathesis occur concurrently. The molecular weight of the isomerized diesters thus varies by multiples of 14 g/mol (one methylene group).

**Figure 5:**
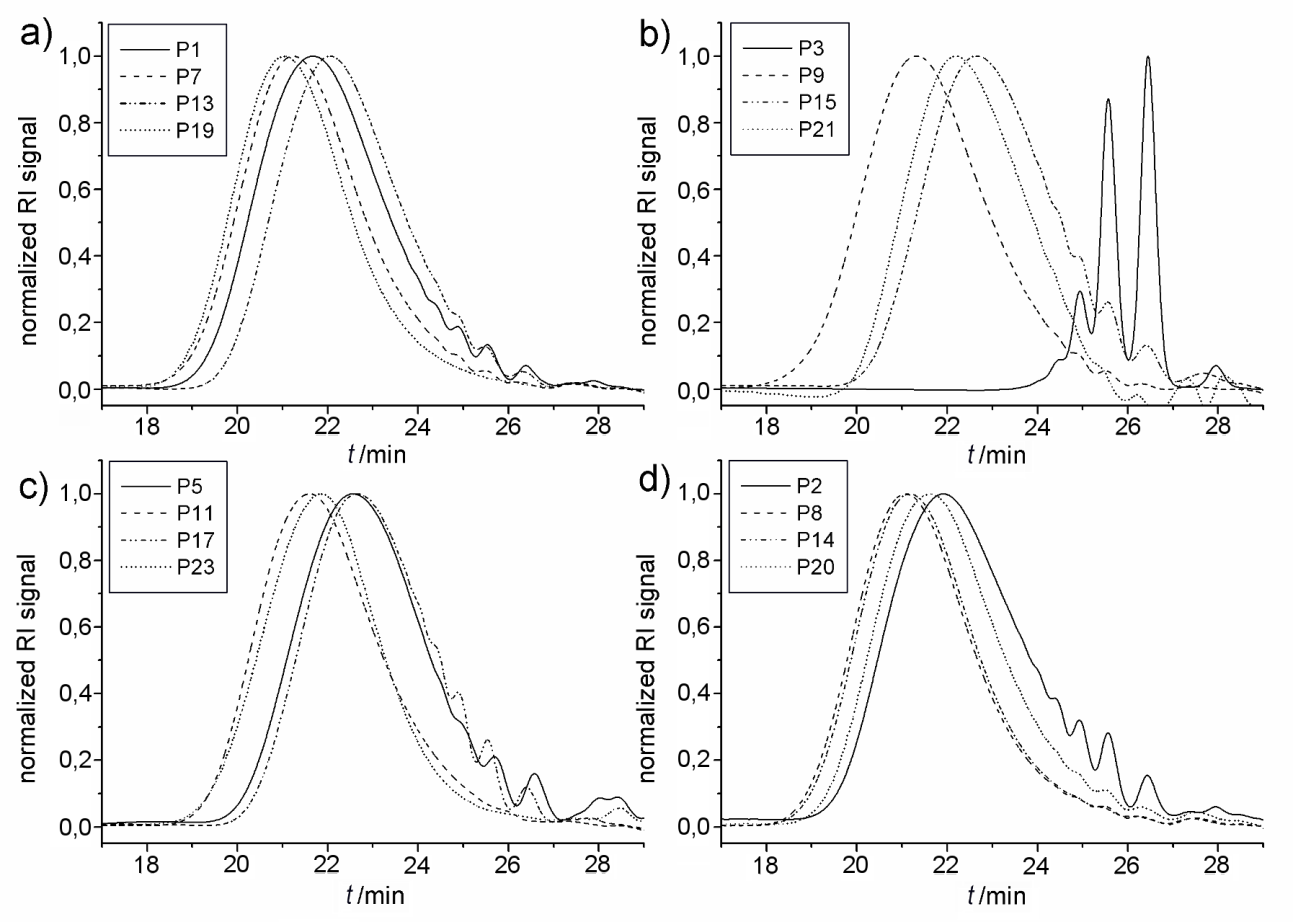
GPC traces of the polymerizations performed at 60, 80, 100 and 120 ºC in presence of a) 0.5 mol % **C1**, b) 0.5 mol % **C2**, c) 0.5 mol % **C3**, and d) 0.5 mol % **C1** with 1 mol % **BQ**.

The analytical data of the polymers synthesized is summarized in Table 1 and Table 2 and selected GPC traces are depicted in Figure 5. Except for the cases in which only oligomers were obtained, monomer conversion was quantitative as determined by the total disappearance of the monomer signal in the GPC traces of the reaction mixtures. The runs at 60 °C showed that, among **C1**, **C2** and **C3** (compare entries 1, 3 and 5 in Table 1, respectively; and Figure 4), **C1** led to the highest molecular weight of around 10 kDa, with a moderate isomerization degree of 36.3% (Table 1, entry 1). Interestingly at this temperature, **C2** showed a considerably lower degree of isomerization of 9.91%; however only oligomers (*M*_n_ 1700 Da) were obtained. Another goal of this research was to suppress the isomerization side reaction and thus to synthesize well-defined polyesters. Benzoquinones are very effective additives for the prevention of the olefin isomerization [21]. Thus, we performed the same set of experiments in the presence of **BQ**, and observed that the degree of isomerization was significantly reduced for **C1,** from 36.3% to 0.7%**.** However, this decrease in the degree of isomerization was accompanied with reduced molecular weights for all studied catalysts. In the worst case of **C2**, the molecular weight was reduced by a factor of 3 (compare entries 3 and 4 in Table 1).

When the polymerization temperature was increased to 80 °C, higher molecular weight polymers were obtained with all the studied catalysts. For instance, **C2** produced a polymer with more than double the molecular weight when increasing the reaction temperature from 60 to 80 °C. Furthermore, the increase of the temperature led to an increase in the amount of isomerization for all of the catalysts. Concerning the isomerization inhibition effect of **BQ** on the catalyst, the effect was significant (factor of 1.5) for **C2** and **C3**, whereas **BQ** was ineffective in the presence of **C1**. In case of **C3**, the molecular weights of the corresponding polymers synthesized with and without **BQ** were similar, with a lower degree of isomerization for **P12**, as expected. Surprisingly, **C1** showed a higher degree of isomerization in the presence of **BQ** at 80 °C.

In an attempt to further increase the molecular weights of the obtained polyesters, all catalysts were also investigated at 100 °C (Table 2). Surprisingly, this further increase of the polymerization temperature led to lower molecular weights for all the studied catalysts. Quite interestingly, at that temperature the most significant inhibition effect of **BQ** on the degree of isomerization was observed for **C2** (compare entries 15 and 16 in Table 2), however, only oligomers were produced. Similarly as for the results at 80 °C, when we used **C1** and **BQ**, we observed an increase of the degree of isomerization along with similar *M*_n_ values (Table 2, entries 13 and 14). On the other hand, **C3** showed the same tendency as at 80 °C. The obtained polymers were less isomerized and had quite high molecular weights. The latter results with **C3** are in good agreement with the results previously obtained for the structurally similar **C4** [30].

**Figure 6:**
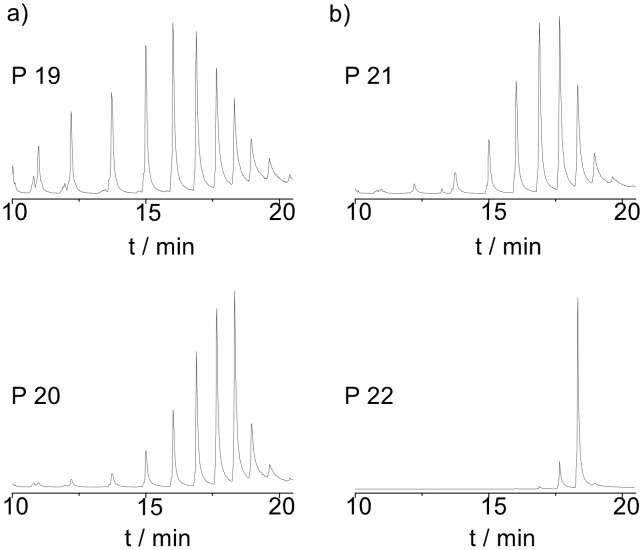
GC-MS study of the acid-catalyzed degradation products of polymers **P19**, **P20**, **P21**, and **P22**.

Furthermore, the catalysts **C1**, **C2,** and **C3,** together with **C4** for comparison, were investigated at 120 °C (Table 2, entries 19, 21, 23, and 25). All complexes provided comparatively high molecular weights, following the order **C1** (~17000 Da) > **C2** (13000 Da) > **C3** (12200 Da) > **C4** (10500 Da). Regardless of the catalyst, all the polymers at that temperature possessed high isomerization values. Subsequently, we tried to reduce the amount of isomerization by performing the same set of reactions in the presence of **BQ** (Table 2, entries 20, 22, 24 and 26). The degree of isomerization was slightly reduced when using **C1** (Figure 6a), and the most prominent effect of **BQ** was observed again for **C2** (Figure 6b); however, this time the polymerization in the presence of **BQ** resulted in polymer with *M*_n_ of 8.5 kDa, compared to the results at lower temperatures. Interestingly, the polymerization with **C3** in the presence of **BQ** followed the same tendency as at 100 °C and resulted in higher molecular weight polymers in comparison to the polymerization without **BQ**, whereas the isomerization remained high (Table 2, entries 23 and 24).

In a previous work, we reported that Hoveyda–Grubbs 2^nd^ generation catalyst (**C4**) yields polymers with molecular weights *M*_n_ of 8000 Da at 80 °C, and 8800 Da at 100 ºC. The isomerization degrees were found to be 24% and 20%, respectively [30]. Herein we have demonstrated that **C4** can be used at a higher temperature (120 °C), in the presence of **BQ** (1 mol %), and with a low amount of catalyst (0.5 mol %), to yield a polymer with *M*_n_ of 12000 Da. However, at 120 °C the amount of isomerization was high with and without **BQ** (entries 25 and 26, Table 2). These results, along with the results discussed in our previous work, clearly show that **C4** can be used in a quite broad temperature range. Interestingly, **BQ** has a more pronounced effect in terms of isomerization inhibition, when compared to the structurally similar **C3** over the whole temperature range studied.

**Figure 7:**
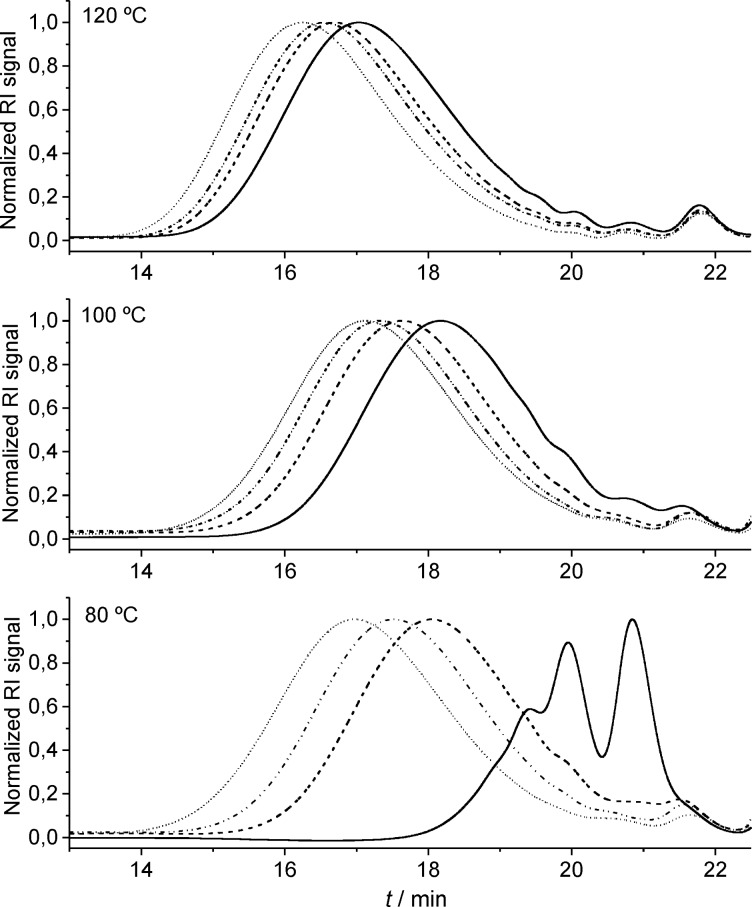
GPC traces of polymerizations performed with **C1** at 80, 100, and 120 ºC. Samples taken at 5 min (―–), 15 min (–), 30 min (– • •), and 120 min (-).

In summary, the tendency found for the activity of these catalysts as a function of the temperature was not linear. A clear increase in the activities was observed on increasing the temperature from 60 ºC to 80 ºC, however, when the temperature was increased to 100 ºC a general activity decrease was observed for all the catalysts, and finally the activity increased again when performing the reactions at 120 ºC. As the temperature is increased the activity of the catalyst increases, however, its degradation might also be accelerated. At 100 ºC, the degradation of the catalyst could be predominant, thus resulting in lower molecular weights. On the other hand, when the temperature is raised to 120 ºC, the catalysts degradation could be compensated by an extremely fast initiation and short-term propagation promoted by the high temperature, giving as a result high molecular weight polymers before degradation of the catalysts occurs. This argumentation is speculative, but in order to provide some data to support this idea, the progress of the polymerization was examined at different times for **C1** at 80, 100 and 120 ºC. Samples were taken at 5, 15, 30, and 120 minutes for each temperature and analyzed by GPC (Figure 7). As predicted from the arguments above, the propagation observed for the polymerization at 80 ºC was slower than that at 100 ºC at short times, however, the polymerization stalled at 100 ºC, possibly due to catalyst degradation, yielding lower molecular weights. Furthermore, the propagation in the initial steps for the polymerization at 120 ºC was found to be the fastest, leading to high molecular weight species in short times before catalyst degradation became predominant.

**Figure 8:**
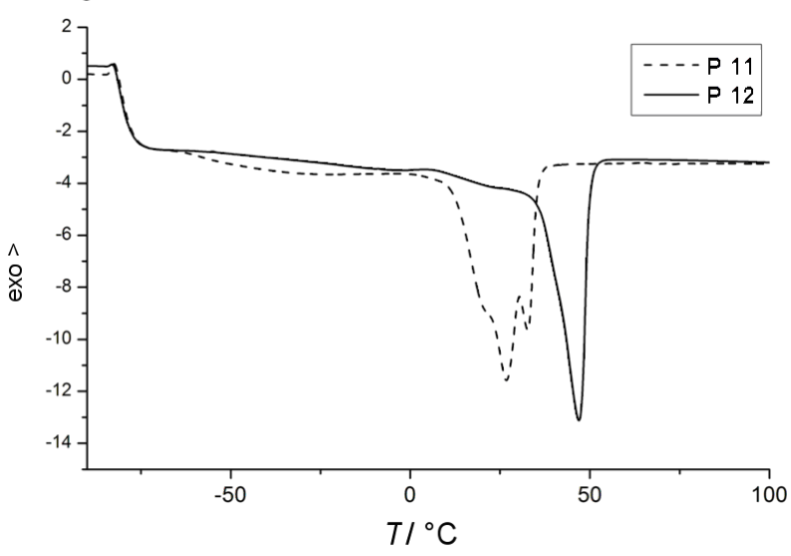
DSC traces of ADMET polymers **P11** and **P12** (Table 1, entries 11 and 12, respectively).

Olefin isomerization occurring during ADMET polymerization leads to macromolecules with ill-defined structures. Depending on the degree of isomerization, the physical properties of the polymers are correspondingly affected. A different insight into the effect of the isomerization ratio on the thermal properties of the polymers can be achieved by differential scanning calorimetry (DSC) analysis of the synthesized polymers. The thermal behavior of two polymers with similar *M*_n_, synthesized at same temperature with and without **BQ**, was studied by DSC (Figure 8). Polymer **P12** (Table 1, entry 12), possessing a lower degree of isomerization, exhibited a quite sharp *T*_m_ peak at 47 °C. On the other hand, the DSC trace of polymer **P11** (Table 1, entry 11), with higher isomerization degree, presented multiple peak melting transitions at lower temperatures resulting from its ill-defined repeat unit structure. These results show that, even if the addition of **BQ** does not completely avoid isomerization in most of the presented examples, polymers with a higher structural regularity can be obtained by with **BQ**.

## Conclusion

The indenylidene Ru-complexes provided an attractive alternative to the benzylidene compounds and allowed polyesters of up to 17000 Da via ADMET polymerization to be prepared, even at elevated temperatures with enhanced activity. Unfortunately, the attempt to synthesize regular polymer architectures by the addition of **BQ** to supress the isomerization reaction, was rather unsuccessful. Nevertheless, the results presented should be regarded as a first experimental data set on these catalysts and further improvement, building on these results, can be expected in the future.

## Experimental

### Materials

10-undecenoic acid (Sigma–Aldrich, 98%), 1,3-propanediol (Sigma–Aldrich, 99.6%), *p*-toluenesulfonic acid monohydrate (Sigma–Aldrich, 98.5%), ethyl vinyl ether (Sigma–Aldrich, 99%), sulfuric acid (Fluka, 95–97%), *p*-benzoquinone (Fluka, 98%), (1,3-bis(2,4,6-trimethylphenyl)-2-imidazolidinylidene) dichloro-(3-phenyl-1*H*-inden-1-ylidene)(tricyclohexylphosphine) ruthenium(II) (Umicore, **C1**), (1,3-bis(2,4,6-trimethylphenyl)-2-imidazolidinylidene)dichloro-(3-phenyl-1*H*-inden-1-ylidene) (pyridyl) ruthenium(II) (Umicore, **C2**), (1,3-bis(2,4,6-trimethylphenyl)-2-imidazolidinylidene)dichloro(2-(1-methylacetoxy)phenyl]methylene ruthenium(II) (Umicore, **C3**), (1,3-bis(2,4,6-trimethylphenyl)-2-imidazolidinylidene)dichloro(*o*-isopropoxyphenylmethylene) ruthenium(II) (Hoveyda–Grubbs catalyst 2^nd^ generation, **C4**, Sigma**–**Aldrich).

#### General Methods

Thin layer chromatography (TLC) was performed on silica gel TLC-cards (layer thickness 0.20 mm, Fluka). Compounds were visualized by permanganate reagent. For column chromatography silica gel 60 (0.035–0.070 mm, Fluka) was used.

^1^H NMR spectra were recorded in CDCl_3_ on Bruker AVANCE DPX spectrometers operating at 300 and 500 MHz. Chemical shifts (δ) are reported in parts per million relative to the internal standard tetramethylsilane (TMS, δ = 0.00 ppm). For the analysis of the polymers the relaxation time was set to 5 seconds.

Mass spectra (ESI) were recorded on a VARIAN 500-MS ion trap mass spectrometer with the TurboDDS^TM^ option installed. Samples were introduced by direct infusion with a syringe pump. Nitrogen served both as the nebulizer gas and the drying gas. Helium was used as cooling gas for the ion trap and collision gas for MS^n^. Nitrogen was generated by a nitrogen generator Nitrox from Dominick Hunter.

GC-MS (EI) chromatograms were recorded with a Varian 431-GC instrument with a capillary column FactorFourTM VF-5ms (30 m × 0.25 mm × 0.25 μm) and a Varian 210-MS detector. Scans were performed from 40 to 650 m/z at a rate of 1.0 scans × s^−1^. The oven temperature was programmed as follows: initial temperature 95 °C, hold for 1 min, ramp at 15 °C × min^−1^ to 200 °C, hold for 2 min, ramp at 15 °C × min^−1^ to 325 °C, hold for 5 min. The injector transfer line temperature was set to 250 °C. Measurements were performed in the split–split mode (split ratio 50:1) with helium as carrier gas (flow rate 1.0 ml × min^−1^).

Polymer molecular weights were determined with an SEC System LC-20 A from Shimadzu equipped with a SIL-20A auto sampler, three PSS SDV columns (5 µm, 300 mm × 7.5 mm, 100 Å, 1000 Å, 10000 Å), and a RID-10A refractive index detector in THF (ﬂow rate 1 mL × min^−1^) at 50 °C. All determinations of molar mass were performed relative to PMMA standards (Polymer Standards Service, *M*_p_ 1100–981.000 Da).

Differential scanning calorimetry (DSC) experiments were carried out under a nitrogen atmosphere at a heating rate of 10 °C × min^−1^ with a DSC821e (Mettler Toledo) calorimeter up to a temperature of 150 °C with a sample mass of approximately 4 mg. The melting temperature, *T*_m_, was recorded as the peak of the endotherm on the second heating scan unless annealing was used as a pretreatment.

#### Synthesis of 1,3-propylene diundec-10-enoate (1)

10-Undecenoic acid (50.00 g, 0.27 mol), 1,3-propanediol (8.4 g, 0.11 mol) and *p*-toluensulfonic acid (3 g, 0.0157 mol) were placed in a round-bottomed flask provided with a magnetic stirrer and a Dean-Stark apparatus. Toluene (200 mL ) was added and the resulting reaction mixture heated to reflux. Water was collected as the reaction proceeded and once the reaction was completed, the reaction mixture was allowed to cool. Toluene was removed under reduced pressure and the residue was filtered through a short pad of basic aluminium oxide with hexane as eluent. After removing the hexane, the crude product was dissolved in diethyl ether (200 mL) and washed two times with water (200 mL). The organic fraction was dried over anhydrous MgSO_4_ and the solvent removed under reduced pressure. The desired product was isolated in 87% yield (39 g).

^1^H NMR (CDCl_3_): δ = 5.85–5.76 (m, 2H, 2x-C*H*=CH_2_), 5.00–4.91 (m, 4H, 2xCH=C*H_2_*), 4.15 (t, 4H, J=6.1 Hz, 2xC*H_2_*OCO-), 2.30 (t, 4H, *J* = 7.3 Hz, C*H_2_*COO-), 2.00 (m, 4H, 2xC*H_2_*-CH=CH2), 1.99–1.94 (m, 2H, *J* = 6.1 Hz, C*H_2_*CH_2_OCO-), 1.64–1.58 (m, 4H, 2xC*H_2_*CH_2_COO-), 1.38–1.34 (m, 4H, 2xC*H_2_*) 1.29–1.24 (br.s, 16H, 2x[4C*H_2_*]) ppm. ^13^C NMR (CDCl3): δ = 173.6 (s, -*C*OO-), 139.0 (s, -*C*H=CH_2_), 114.1 (s, -CH=*C*H_2_), 60.7 (s, *C*H_2_OCO-), 34.1 (s, *C*H_2_), 33.7 (s, *C*H_2_), 29.2 (s, *C*H_2_), 29.1 (s, *C*H_2_), 29.0 (s, *C*H_2_), 28.8 (s, *C*H_2_), 24.8 (s, CH_2_) ppm. MS (EI): *m/z* = 408 [M]^+^, calc. 408.3239.

#### ADMET polymerization (P1-P26)

To 1 g (2.45 mmol) of 1,3-propylene diundec-10-enoate in a tube equipped with a screw, 0.5 mol % of the corresponding ruthenium catalyst, (**C1**: 11.6 mg (0.0122 mmol), **C2**: 9.1 mg (0.0122 mmol), **C3**: 8 mg (0.0122 mmol) and **C4**: 7.7 mg) was added at the desired reaction temperature (60–120 °C). In some cases, 1 mol % of **BQ** was added to the reaction mixture 10 min before the addition of the catalyst. Reactions were carried out in parallel using a carousel reaction station from Radleys. Stirring was continued at the selected temperature under a continuous flow of nitrogen for 5 h. After 5 h reaction time, the reaction mixture was dissolved in 1 mL of THF and polymerization halted by the addition of 1 mL of ethyl vinyl ether. The mixture was then stirred for 30 min at room temperature. The crude product was purified by precipitation into cold methanol. Final polymer molecular weights were determined after precipitation with the above mentioned GPC system.

#### Transesterification of the obtained polymers (P1-P26) and GC-MS analysis

The respective polymer (30 mg), excess methanol (4 mL) and concentrated sulfuric acid (5 drops) were added to a carousel reaction tube, stirred magnetically, and refluxed at 85 °C for 5 h. At the end of the reaction, the excess of methanol was removed under reduced pressure. The residue was then dissolved in diethyl ether and filtered through a small column of basic aluminium oxide. Subsequently, GC-MS samples were prepared by taking 500 µL of this solution and diluting it with methanol (500 µL). The percentage of olefin isomerization was calculated based on peak areas of the isomerized diesters.
